# COVID-19 and Undiagnosed Pre-diabetes or Diabetes Mellitus Among International Migrant Workers in Singapore

**DOI:** 10.3389/fpubh.2020.584249

**Published:** 2020-11-11

**Authors:** Louis Y. Tee, Sharifah Munirah Alhamid, Jeriel L. Tan, Theik Di Oo, Jaime Chien, Primavera Galinato, Seow Yen Tan, Shafi Humaira, Raymond Kok Choon Fong, Troy H. Puar, Wann Jia Loh, Anindita Santosa, Joan Khoo, Barbara Helen Rosario

**Affiliations:** ^1^Department of Geriatric Medicine, Changi General Hospital, Singapore, Singapore; ^2^Department of Infectious Diseases, Changi General Hospital, Singapore, Singapore; ^3^Department of Endocrinology, Changi General Hospital, Singapore, Singapore; ^4^Department of Rheumatology, Changi General Hospital, Singapore, Singapore

**Keywords:** COVID-19, diabetes mellitus, impaired glucose tolerance (IGT), international migrant worker, pre-diabetes (pre-DM)

## Abstract

**Objective:** Migrant workers, a marginalized and under-resourced population, are vulnerable to coronavirus disease 2019 (COVID-19) due to limited healthcare access. Moreover, metabolic diseases—such as diabetes mellitus (DM), hypertension, and hyperlipidemia—predispose to severe complications and mortality from COVID-19. We investigate the prevalence and consequences of undiagnosed metabolic illnesses, particularly DM and pre-diabetes, in international migrant workers with COVID-19.

**Methods:** In this retrospective analysis, we analyzed the medical records of international migrant workers with laboratory-confirmed COVID-19 hospitalized at a tertiary hospital in Singapore from April 21 to June 1, 2020. We determined the prevalence of DM and pre-diabetes, and analyzed the risk of developing complications, such as pneumonia and electrolyte abnormalities, based on age and diagnosis of DM, and pre-diabetes.

**Results:** Two hundred and fouty male migrant workers, with mean age of 44.2 years [standard deviation (SD), 8.5years], were included. Twenty one patients (8.8%) were diagnosed with pre-diabetes, and 19 (7.9%) with DM. DM was poorly controlled with a mean HbA1c of 9.9% (SD, 2.4%). 73.7% of the patients with DM and all the patients with pre-diabetes were previously undiagnosed. Pre-diabetes was associated with higher risk of pneumonia [odds ratio (OR), 10.8, 95% confidence interval (CI), 3.65–32.1; *P* < 0.0001], hyponatremia (OR, 8.83; 95% CI, 1.17–66.6; *P* = 0.0342), and hypokalemia (OR, 4.58; 95% CI, 1.52–13.82; *P* = 0.0069). Moreover, patients with DM or pre-diabetes developed COVID-19 infection with lower viral RNA levels.

**Conclusions:** The high prevalence of undiagnosed pre-diabetes among international migrant workers increases their risk of pneumonia and electrolyte abnormalities from COVID-19.

## Introduction

There are 164 million international migrant workers globally, making them the world's largest transnational migrant population (1). Migrant workers face systemic injustices that lead to limited access to healthcare, such as segregated housing, inadequate education, and socioeconomic deprivation (1–4). Moreover, effective healthcare policies designed to protect this vulnerable patient group are challenging to formulate because data regarding the health outcomes of this neglected population are scarce and fragmented (1). Therefore, migrant workers are at a heightened risk of transmission of coronavirus 2019 (COVID-19) (5).

Indeed, as of July 2, 2020, there were 41,646 migrant workers living in dormitories diagnosed with COVID-19 in Singapore, making up 94.4% of the 44,122 cases of COVID-19 in the country (6). As of December 2019, there were 1.4 million international migrant workers in Singapore (7). Close to 1 million entered on work permits for low-wage jobs, such as 293,300 male construction workers who live in dormitories (7), and previous studies have found that these migrant workers are especially susceptible to outbreaks of infectious diseases such as malaria, enteric fevers, viral hepatitis, and tuberculosis compared to residents (4). With large numbers of migrant workers admitted to the healthcare system for COVID-19, this situation has provided a unique opportunity to evaluate the general health of this hidden population. In the course of caring for these migrant workers, physicians searched for potential complications of COVID-19 infections, and screened for common chronic diseases, such as diabetes mellitus, which may exacerbate COVID-19 infections.

Emerging evidence from the COVID-19 pandemic have demonstrated that hyperglycemia on admission to a hospital portends increased severity of disease and higher mortality (8, 9). While diabetes mellitus (DM) is a risk factor for severe COVID-19 (10–13), hyperglycemia itself, and not just a known history of DM, have been shown to be associated with worse prognosis (8, 9). It has been proposed that this is because glycosylation of the spike protein of severe acute respiratory syndrome coronavirus 2 (SARS-CoV-2), the virus that causes COVID-19, modulates its binding to the angiotensin converting enzyme receptor 2 (ACE2) on host tissue (14). Since increased glycosylation of SARS-CoV-2 and ACE2 promotes the entry of SARS-CoV-2 into host cells, it has been postulated that higher blood glucose levels would lead to greater susceptibility to COVID-19 infection and amplified disease severity (15). Moreover, SARS-CoV-2 binds to tissues with high ACE2 expression, such as the lungs, liver, kidneys and blood (16), and in addition to pneumonia, COVID-19 has been reported to cause systemic complications such as electrolyte abnormalities (17), thrombocytopenia (18), anemia (19), leukopenia (20), and transaminitis (21). In fact, it has been proposed that screening for hyperglycemia and prompt blood glucose control should be mandatory for all COVID-19 cases to improve prognosis (22). Even though current guidelines suggest routine screening for DM and pre-diabetes (also known as impaired glucose tolerance) for adults aged 40 years and older (23), underdiagnosis is common among Singapore residents (24). Among Singapore residents, the prevalence of DM is 8.6%, of which a third are unaware they have the disease (25). We hypothesized that the prevalence of undiagnosed DM and pre-diabetes will be higher among international migrant workers compared to Singapore residents due to their limited access to health care, and that this will lead to more complications of COVID-19.

## Methods

### Inclusion Criteria

This retrospective cohort study was performed in a cohort of adult migrant workers diagnosed with COVID-19. The patients were admitted to one of the three COVID-19 cohort wards available in Changi General Hospital, a tertiary hospital in Singapore, from April 21 to June 1, 2020. Patients admitted to this ward were cared for by physicians from the Department of Geriatric Medicine with regular advice from the Department of Infectious Diseases and were selected for admission based on male sex, hemodynamic stability, and no requirement for supplemental oxygen. Inclusion criteria for the analysis were age of 21 years old and above, a confirmed laboratory diagnosis of COVID-19 infection using SARS-CoV-2 reverse-transcriptase polymerase-chain reactions (RT-PCR) from nasopharyngeal swabs and employment outside the country of origin. The demographic data collected from their electronic medical records were age, sex, ethnicities, and past medical histories. Additionally, hemodynamic parameters (blood pressure, BP; and heart rate, HR) on admission and discharge; new diagnoses of chronic diseases made during hospitalization (such as hypertension, diabetes mellitus, and hyperlipidemia); and hematological, biochemical, and radiological abnormalities were extracted from the medical records.

### Diagnosis and Treatment of Chronic Diseases

Patients were screened for diabetes mellitus with HbA1c or OGTT if random blood glucose was elevated. Diagnosis of diabetes mellitus and pre-diabetes was based on the American Diabetic Association 2019 guidelines (23). BP was monitored closely throughout admission. The diagnostic criterion for hypertension was based on the Eighth Joint National Committee (JNC 8) guidelines (26). Screening for hyperlipidemia was done if there were clinical risk factors or presence of other cardiovascular risk factors (27). Patients were deemed tachycardic if HR was more than 100 beats per minute (beats/min). Hyperlipidemia was diagnosed according to the American Heart Association guidelines (27). Appropriate and prompt treatment was started for newly diagnosed DM, such as metformin, glipizide and/or linagliptin; and pre-meal capillary blood glucose measurements with subcutaneous sliding-scale insulin.

### Statistics

Statistical analysis was performed using SPSS 25 software. Statistical significance, determined with two-tailed, unpaired testing, was evaluated at the 0.05 level. For continuous variables (such as age, Ct values, blood pressures, and heart rates), two-tailed t-tests were used to compare the means of two groups, and single-factor ANOVA was used to compare the means of three or more groups. For categorical variables (such as the proportion of patients with complications like pneumonia), the chi-square test was used to compare the proportions between groups. Odds ratios were calculated using binomial logistic regression. There was no funding obtained specifically for this study. Ethics review was overseen by the Singapore Heath System (SingHealth) Centralized Institutional Review Board (CIRB). All data was anonymized and irreversibly de-identified to protect patient privacy.

## Results

### Patient Characteristics

Two hundred and fouty four patients' electronic medical records were reviewed. Three patients were Singapore residents and one patient was younger than 21 years old, and their records were excluded from the analysis. The medical records of 240 patients were included in our study. The demographic data were collated and analyzed based on age (Table 1) and diagnosis of DM or pre-diabetes (Table 2). All the patients were male, and the mean age was 44.2 years [standard deviation (SD), 8.5 years]. Our population consists of the following ethnicities: Bangladeshi (*n* = 86, 35.8%), Indian (*n* = 70, 29.2%), Chinese (*n* = 66, 27.5%), Thai (*n* = 12, 5.0%), and other ethnicities (*n* = 6, 2.5%). The mean cycle threshold (Ct) value for the N gene was 23.9 (SD, 8.3), and the mean Ct value for the E gene was 23.6 (SD, 8.1). The Ct value was significantly higher in patients with DM (N gene Ct value, 30.7; SD, 4.9 and E gene Ct value, 30.8; SD, 5.1) and pre-diabetes (N gene Ct value, 25.8; SD, 7.5 and E gene Ct value, 26.8; SD, 7.5), suggesting that lower viral RNA levels resulted in COVID-19 infection. On admission, 23 patients (9.6%) were asymptomatic, while the rest had symptoms of rhinorrhea, sore throat, cough, dyspnea, pleuritic chest pain, diarrhea, lethargy, and myalgia.

**Table 1:** Demographics and clinical characteristics, stratified by age.

	**All ages** **(*n* = 240)**	**20 to 29 years** **(*n* = 20)**	**30 to 39 years** **(*n* = 39)**	**40 to 49 years** **(*n* = 120)**	**≥ 50 years** **(*n* = 61)**	**P value**
Age, mean (SD), years	44.2 (8.5)	26.2 (2.1)	33.6 (2.8)	46.4 (2.1)	52.7 (2.8)	<0.0001
**Ethnicity**						
Bangladeshi, *n* (%)	86 (35.8)	16 (80.0)	23 (59.0)	38 (31.7)	9 (14.8)	0.0115
Indian, *n* (%)	70 (29.2)	4 (20.0)	14 (36.0)	33 (27.5)	19 (31.2)	
Chinese, *n* (%)	66 (27.5)	0 (0.0)	2 (5.1)	43 (35.8)	21 (34.4)	
Thai, *n* (%)	12 (5.0)	0 (0.0)	0 (0.0)	3 (2.5)	9 (14.8)	
Others, *n* (%)	6 (2.5)	0 (0.0)	0 (0.0)	3 (2.5)	3 (4.9)	
**Co-morbidity**						
Diabetes mellitus, *n* (%)	19 (7.9)	0 (0.0)	1 (2.6)	12 (10.0)	6 (9.8)	0.0057
Hypertension, *n* (%)	18 (7.5)	0 (0.0)	3 (7.7)	7 (5.8)	8 (13.1)	0.0071
Hyperlipidemia, *n* (%)	11 (4.6)	0 (0.0)	2 (5.1)	4 (3.3)	5 (8.2)	0.0495
**SARS-CoV-2 RT-PCR statistics**						
N gene Ct value, mean (SD)	23.9 (8.3)	17.7 (7.3)	19.0 (8.0)	25.8 (7.7)	27.2 (6.8)	<0.0001
E gene Ct value, mean (SD)	23.6 (8.1)	17.5 (6.7)	19.2 (8.5)	25.4 (7.5)	26.8 (6.6)	<0.0001
**Hemodynamic parameters on admission**						
Systolic BP, mean (SD), mmHg	131 (17)	122 (11)	128 (16)	130 (15)	138 (20)	0.0004
Diastolic BP, mean (SD), mmHg	84 (10)	79 (8)	80 (11)	84 (9)	87 (9)	0.0009
Heart rate, mean (SD), beats/min	88 (13)	87 (14)	84 (11)	88 (13)	89 (14)	0.2920
**Hemodynamic parameters on discharge**						
Systolic BP, mean (SD), mmHg	121 (12)	115 (9)	119 (12)	122 (12)	121 (13)	0.8375
Diastolic BP, mean (SD), mmHg	80 (9)	78 (8)	77 (8)	80 (9)	79 (8)	0.3944
Heart rate, mean (SD), beats/min	82 (12)	79 (13	80 (12)	83 (12)	82 (12)	0.3303
Serum glucose, mean (SD), mmol/L	7.5 (3.0)	6.4 (1.7)	6.6 (2.6)	7.7 (3.5)	7.4 (2.2)	0.3877
Length of stay, mean (SD), days	5.3 (2.9)	4.3 (1.8)	5.2 (4.0)	5.5 (3.0)	5.3 (2.1)	0.4353
**Discharge destination**						
Home, *n* (%)	47 (19.6)	1 (5.0)	4 (10.3)	31 (25.8)	11 (18.0)	0.1835
Isolation facility, *n* (%)	167 (69.6)	19 (95.0)	35 (89.7)	71 (59.2)	42 (68.9)	
Subacute hospital, *n* (%)	27 (11.2)	0 (0.0)	0 (0.0)	18 (15.0)	9 (14.8)	
Re-admission to hospital, *n* (%)	7 (2.92)	0 (0.0)	2 (5.13)	4 (3.33)	1 (1.64)	0.1569

*Data are mean (standard deviations) and number of patients (percentage of total number of patients in each age group). P-values are calculated using single-factor ANOVA and chi–square tests. RT-PCR, reverse-transcriptase polymerase-chain reaction; Ct, cycle threshold; BP, blood pressure*.

**Table 2:** Demographics and clinical characteristics, stratified by diagnosis of diabetes mellitus, and pre-diabetes.

	**All** **(*n* = 240)**	**Diabetes mellitus** **(*n* = 19)**	**Pre-diabetes** **(*n* = 21)**	**Normal glucose control** **(*n* = 200)**	***P*-value**
Age, mean (SD), years	44.2 (8.5)	47.3 (4.7)	47.7 (7.8)	43.5 (8.7)	0.0248
**Ethnicity**					
Bangladeshi, *n* (%)	86 (35.8)	7 (36.8)	2 (9.5)	77 (38.5)	1.0000
Indian, *n* (%)	70 (29.2)	9 (47.4)	6 (28.6)	55 (27.5)	
Chinese, *n* (%)	66 (27.5)	2 (10.5)	12 (57.1)	52 (26.0)	
Thai, *n* (%)	12 (5.0)	1 (5.3)	1 (4.8)	10 (5.0)	
Others, *n* (%)	6 (2.5)	0 (0.0)	0 (0.0)	6 (3.0)	
**Co-morbidity**					
Hypertension, *n* (%)	18 (7.5)	2 (10.5)	3 (14.3)	13 (6.5)	0.1058
Hyperlipidemia, *n* (%)	11 (4.6)	3 (15.8)	4 (19.0)	4 (2.0)	0.0005
**SARS-CoV-2 RT-PCR statistics**					
N gene Ct value, mean (SD)	23.9 (8.3)	30.7 (4.9)	25.8 (7.5)	23.2 (8.3)	0.0062
E gene Ct value, mean (SD)	23.6 (8.1)	30.8 (5.1)	26.8 (7.5)	22.6 (8.0)	0.0006
**Hemodynamic parameters on admission**					
Systolic BP, mean (SD), mmHg	131 (17)	147 (21)	134 (8)	129 (16)	<0.0001
Diastolic BP, mean (SD), mmHg	84 (10)	92 (9)	85 (8)	83 (10)	<0.0001
Heart rate, mean (SD), beats/min	88 (13)	97 (14)	91 (16)	86 (13)	0.0017
**Hemodynamic parameters on discharge**					
Systolic BP, mean (SD), mmHg	121 (12)	127 (14)	120 (9)	120 (13)	0.0678
Diastolic BP, mean (SD), mmHg	80 (9)	82 (8)	81 (7)	78 (9)	0.1131
Heart rate, mean (SD), beats/min	82 (12)	86 (11)	84 (11)	82 (12)	0.3197
Serum glucose, mean (SD), mmol/L	7.5 (3.0)	13.8 (5.0)	9.6 (1.5)	6.4 (1.4)	<0.0001
Length of stay, mean (SD), days	5.3 (2.9)	6.3 (2.3)	6.0 (3.1)	5.1 (2.9)	0.1340
**Discharge destination**					
Home, *n* (%)	47 (19.6)	4 (21.1)	4 (19.0)	39 (19.4)	0.9594
Isolation facility, *n* (%)	167 (69.6)	14 (73.7)	14 (66.7)	139 (69.2)	
Subacute hospital, *n* (%)	27 (11.2)	1 (5.3)	3 (14.3)	23 (11.4)	
Re-admission to hospital, *n* (%)	7 (2.92)	1 (5.3)	0 (0.0)	6 (3.0)	0.5768

*Data are mean (standard deviations) and number of patients (percentage of total number of patients in each group). P-values are calculated by comparing three groups (diabetes mellitus, pre-diabetes, and normal glucose control) using single-factor ANOVA and chi–square tests. RT-PCR, reverse-transcriptase polymerase-chain reaction; Ct, cycle threshold; BP, blood pressure*.

### Screening for DM and Pre-diabetes

The prevalence of DM in our cohort is 7.9% (19 of 240 patients), of which 73.7% (14 of 19 patients with diabetes) were newly diagnosed. The mean serum glucose on admission, measured in 201 patients, was 7.5 mmol/L (SD, 3.0 mmol/L). Random plasma glucose was >7.8 mmol/L in 64 patients (31.8%), of which 59 (24.5%) were not previously known to have DM. Thirty two patients underwent OGTT, and HbA1c levels were measured in 45 patients. From the OGTT and/or HbA1c results, 14 patients (5.8%) were newly diagnosed to have DM. All the patients with DM had an HbA1c >7.0%, with a mean HbA1c of 9.9% (SD, 2.4%). Patients diagnosed with DM were started on appropriate oral hypoglycemic agents such as metformin, glipizide and/or linagliptin, as well as pre-meal capillary blood glucose monitoring with subcutaneous sliding-scale insulin injections. Additionally, 21 patients (8.8%) were newly diagnosed with pre-diabetes, defined as an HbA1c between 5.7 and 6.4% and/or a 2 h serum glucose for the OGTT between 7.8 and 11.0 mmol/L, and they were counseled on lifestyle modifications and advised to follow up with a primary-care physician for development of diabetes or resolution of pre-diabetes. Patients with DM or pre-diabetes were older (DM, 47.3 years; SD, 4.7 vs. pre-diabetes, 47.7 years; SD, 7.8 vs. normal glucose control, 44.2 years; SD, 8.5; *P* = 0.0248), and had a higher prevalence of hyperlipidemia (DM, 15.8% vs. pre-diabetes, 19.0% vs. normal glucose control, 2.0%; *P* = 0.0005; Table 2).

### Hypertension

The prevalence of hypertension in our cohort was 7.5% (18 of 240 patients), of which 61.1% (8 of 18 patients with hypertension) were newly diagnosed. Eighty two patients (34.2%) were hypertensive on admission, with a mean systolic BP of 149 mmHg (SD, 10 mmHg) and a mean diastolic BP of 93 mmHg (SD, 9 mmHg). No patients were in hypertensive urgency, defined as a systolic BP >180 mHg and/or a diastolic BP >120 mmHg. Eleven patients (4.6%) were diagnosed with hypertension because they were persistently hypertensive over 2 to 3 days and were started on anti-hypertensive medications such as amlodipine, atenolol or enalapril. Twenty seven patients (11.3%) were hypertensive on discharge, with a mean systolic BP of 138 mmHg (SD, 10 mmHg) and a mean diastolic BP of 92 mmHg (SD, 4 mmHg). Patients who had transient BP readings above 140/90 mmHg were advised to follow up with a family physician to monitor their BP on discharge. Patients with DM and pre-diabetes had higher systolic (DM; 147 mmHg; SD, 21 vs. pre-diabetes; 134 mmHg; SD, 17 vs. normal glucose control; 129 mmHg; SD, 16; *P* < 0.0001) and diastolic (DM, 93 mmHg; SD, 9 vs. pre-diabetes; 85mmHg; SD 8 vs. normal glucose control; 83 mmHg; SD, 10 mmHg; *P* < 0.0001) blood pressures and heart rates (DM; 97 beats/min; SD, 14 vs. pre-diabetes; 92 beats/min; SD 16 vs. normal glucose control; 86 beats/min; SD, 13; *P* = 0.0017) on admission, but not on discharge (Table 2).

### Hepatic, Electrolyte, and Hematological Abnormalities

Serum sampling was undertaken in 201 patients and the results were analyzed based on age (Table 3) and diagnosis of DM/pre-diabetes (Table 4), and the association of hepatic, electrolyte, and hematological complications with age and/or DM/pre-diabetes was assessed (Table 5). The most common abnormal blood result was deranged liver enzymes with 46 patients (23.4%) affected, of which six (3.6%) had alanine aminotransferase levels >two times the upper limit of normal. The incidence of severe transaminitis was higher in patients with DM (15.8 vs. 1.5%, *P* = 0.0238; Table 4).

**Table 3:** Incidence of laboratory and radiographical abnormalities, stratified by age.

	**All ages**	**20 to 29 years**	**30 to 39 years**	**40 to 49 years**	**≥50 years**	***P*-value**
**Renal panel (*****n*** **=** **201)**						
Hyponatremia, *n* (%)	5 (2.5)	0 (0.0)	0 (0.0)	2 (1.74)	3 (4.9)	0.0557
Hypokalemia, *n* (%)	20 (10.0)	0 (0.0)	2 (10.0)	10 (8.7)	8 (13.1)	0.0111
**Liver function test (*****n*** **=** **197)**						
Transaminitis, *n* (%)	46 (23.4)	3 (75.0)	5 (27.8)	22 (19.3)	16 (26.2)	<0.0001
ALT >2 times ULN, *n* (%)	7 (3.6)	0 (0.0)	0 (0.0)	4 (3.5)	3 (5.0)	0.0527
**Full blood count (*****n*** **=** **201)**						
All disorders, *n* (%)	44 (21.9)	0 (0.0)	3 (15.0)	29 (25.2)	12 (19.6)	<0.0001
Thrombocytopenia, *n* (%)	8 (4.0)	0 (0.0)	0 (0.0)	7 (6.1)	1 (1.6)	0.0151
Leukopenia, *n* (%)	8 (4.0)	0 (0.0)	0 (0.0)	7 (6.1)	1 (1.6)	0.0151
Anemia, *n* (%)	8 (4.0)	0 (0.0)	0 (0.0)	5 (4.4)	3 (4.9)	0.0418
Polycythemia, *n* (%)	4 (2.0)	0 (0.0)	0 (0.0)	2 (1.7)	2 (3.3)	0.1834
Microcytosis, *n* (%)	15 (7.5)	0 (0.0)	3 (15.0)	7 (6.1)	5 (8.2)	0.0015
Macrocytosis, *n* (%)	1 (0.5)	0 (0.0)	0 (0.0)	1 (0.9)	0 (0.0)	0.6213
**Chest X-ray (*****n*** **=** **226)**						
Infective changes, *n* (%)	20 (8.9)	0 (0.0)	2 (6.9)	8 (6.7)	10 (16.4)	0.0010
Atelectasis, *n* (%)	6 (2.7)	0 (0.0)	0 (0.0)	1 (0.8)	5 (8.2)	0.0004
Nodules, granulomata, *n* (%)	5 (2.2)	0 (0.0)	0 (0.0)	5 (4.2)	0 (0.0)	0.0391
Pleural thickening/scarring, *n* (%)	3 (1.3)	0 (0.0)	0 (0.0)	1 (0.8)	2 (3.3)	0.1268

*Data are number of patients (percentage of the total number of patients tested in each group). P-values are calculated by comparing all four age groups using chi–square tests. ALT, alanine aminotransferase; ULN, upper limit of normal*.

**Table 4:** Incidence of laboratory and radiographical abnormalities stratified by diagnosis of diabetes mellitus and pre-diabetes.

	**All**	**Diabetes mellitus**	**Pre-diabetes**	**Normal glucose control**	***P*-value**
**Renal panel (*****n*** **=** **201)**					
Hyponatremia, *n* (%)	5 (2.5)	1 (5.2)	2 (9.5)	2 (1.2)	0.1119
Hypokalemia, *n* (%)	20 (10.0)	1 (5.3)	6 (28.6)	13 (8.0)	0.0236
**Liver function test (*****n*** **=** **197)**					
Transaminitis, *n* (%)	46 (23.4)	7 (36.8)	2 (9.5)	37 (18.5)	0.0631
ALT >2 times ULN, *n* (%)	7 (3.6)	3 (15.8)	1 (4.8)	3 (1.5)	0.0238
**Full blood count (*****n*** **=** **201)**					
Thrombocytopenia, *n* (%)	8 (4.0)	1 (5.3)	2 (9.5)	5 (3.1)	0.2685
Leukopenia, *n* (%)	8 (4.0)	0 (0.0)	3 (14.3)	5 (3.1)	0.0988
Anemia, *n* (%)	8 (4.0)	0 (0.0)	2 (9.5)	6 (3.7)	0.3680
**Chest X-ray (*****n*** **=** **226)**					
Infective changes, *n* (%)	20 (8.9)	2 (10.5)	8 (38.1)	10 (5.4)	0.0001
Atelectasis, *n* (%)	6 (2.7)	0 (0.0)	1 (4.8)	5 (2.7)	0.6604

*Data are number of patients (percentage of the total number of patients tested in each group). P-values are calculated by comparing three groups (diabetes mellitus, pre-diabetes and normal glucose control) using chi–square tests. ALT, alanine aminotransferase; ULN, upper limit of normal*.

**Table 5:** Association between age, diabetes mellitus, and pre-diabetes with complications of COVID-19.

	**Diabetes mellitus**	***P*-value**	**Pre-diabetes**	***P*-value**	**Age ≥50 years**	***P*-value**
Pneumonia, OR (95% CI)	2.07 (0.42–10.2)	0.3783	10.8 (3.65–32.1)	<0.0001	3.13 (1.31–8.41)	0.0116
Hyponatremia, OR (95% CI)	4.67 (0.40–54.3)	0.2194	8.83 (1.17–66.6)	0.0342	3.57 (0.58–21.9)	0.1703
Hypokalemia, OR (95% CI)	0.64 (0.07–5.16)	1.2571	4.58 (1.52–13.82)	0.0069	1.62 (0.63–4.20)	0.3227
Transaminitis, OR (95% CI)	1.88 (0.69–5.11)	0.2206	0.339 (0.08–1.52)	1.2006	1.24 (0.62–2.50)	0.5516

*Data are odds ratios (95% confidence intervals), calculated using binomial logistic regression. Diabetes mellitus and pre-diabetes are compared to normal glucose control (OR = 1). Age 50 and greater is compared to age <50 (OR = 1). OR, odds ratio; CI, confidence interval*.

Hematological abnormalities were also common, and six abnormalities were observed in 44 individual patients (17.9%). Microcytosis was found in 15 patients (7.5%); thrombocytopenia in eight (4.0%); leukopenia in eight (4.0%); anemia in eight (4.0%); polycythemia in four (1.99%); and macrocytosis in one (0.5%). Of the eight patients who were anemic, five had normocytic anemia, two had microcytic anemia, and one had macrocytic anemia. Of the five patients who had the anemia work-up completed, three cases were iron-deficient, one was vitamin-B12 deficient, and one was both iron and vitamin-B12 deficient. One patient had macrocytosis without anemia and was found to be vitamin-B12 deficient.

Lastly, the renal panels revealed electrolyte disturbances, such as hypokalemia in 20 patients (10.0%) with a mean serum potassium of 3.3 mmol/L (SD, 0.2 mmol/L), and hyponatremia in 5 patients (2.5%), with a mean serum sodium of 130 mmol/L (SD, 4 mmol/L). Pre-diabetes was associated with higher risk of hyponatremia [odds ratio (OR), 8.83; 95% confidence interval (CI), 1.17–66.6; *P* = 0.0342] and hypokalemia (OR, 4.58; 95% CI, 1.52–13.8; *P* = 0.0069; Table 5).

### Radiographical Abnormalities

Two hundred and twenty six patients (94.2%) had chest X-rays performed. Twenty patients (8.8%) were found to have infective changes consistent with pneumonia, and six patients (2.7%) had atelectasis. Five patients (2.2%) had lung nodules suggestive of past or active tuberculosis. Further sputum examination did not yield evidence of tuberculosis. Pleural thickening was found in three patients (1.3%), and the patients reported either a significant smoking history or previous occupational exposure to asbestos, albeit while wearing protective equipment. Higher risk of pneumonia was linked to pre-diabetes (OR, 10.8; 95% CI, 3.65–32.1; *P* < 0.0001) and age 50 years or older (OR, 3.13; 95% CI, 1.31–8.41; *P* = 0.0116; Table 5).

### Antibiotics and Hydroxychloroquine

Of the 34 patients with abnormalities on chest X-ray, 15 patients (44.1%) did not receive antibiotics. Eleven patients (32.4%) received oral amoxicillin/clavulanic acid 1 g twice a day for 7 days and azithromycin 500 mg once a day for 3 days. Five patients (14.7%) received amoxicillin/clavulanic acid 1 g twice a day for 5 days and azithromycin 500 mg once a day for 3 days; 2 (5.9%) received amoxicillin/clavulanic acid 1 g twice a day for 7 days and doxycycline 100 mg twice a day for 7 days; and 1 (2.9%) received amoxicillin/clavulanic acid 1 g twice a day for 7 days. Of the 20 patients that had infective changes on their X-rays, 18 (90.0%) received empirical antibiotics, which was indicated for bacterial co-infection.

Of the 240 patients in the cohort, two patients (0.8%) were transferred to another ward due to worsening laboratory and radiological results, where they received oral hydroxychloroquine 400 mg twice a day for 1 day then 200 mg twice a day for 4 days. They did not require supplemental oxygen nor intensive care during their admission and were discharged home. One of the patients had pre-diabetes and the other was not diagnosed with pre-diabetes/DM.

### Lengths of Stay, Discharge Destinations, and Re-admissions

The mean length of stay (LOS) in the acute hospital ward was 5.3 days (SD, 2.9 days), and patients with DM and pre-diabetes were on average hospitalized for 1 day longer (6.1 days; SD, 2.7 days vs. 5.1 days; SD, 2.9 days; *P* = 0.0379). Seven patients (2.9%) were re-admitted to an acute hospital within 30 days of discharge. Two cases (0.8%) were re-admitted for complications of COVID-19, both of whom were not diagnosed with DM or pre-diabetes, while five cases (2.1%) were re-admitted for other reasons. Fouty seven patients (19.6%) were discharged after testing negative for SARS-CoV-2 on two consecutive days. For the rest of the patients, who tested SARS-CoV-2 PCR positive during their admissions, 166 patients (69.2%) were sent to an isolation facility within the community, and 27 patients (11.3%) were transferred to a subacute community hospital, where they were monitored more closely compared to isolation facilities. There were no mortalities at 30 days. Patients with DM and pre-diabetes did not have a significant difference in re-admission rates at 30 days and discharge destination.

### Newly Diagnosed Diseases

Thirteen patients (5.4%) reported that they had a known past medical history on admission. Nine patients reported one co-morbidity and four patients reported multiple co-morbidities, which included type 2 diabetes mellitus in five patients (2.1%), hypertension in seven patients (2.9%), and hyperlipidemia in three patients (1.3%). Other notable past medical issues included work-related injuries (such as hand laceration, blunt trauma to upper limb, and vehicular trauma to knee) in three patients, asthma and ischemic heart disease.

In total, 70 patients (29.2%) in the cohort were diagnosed with either a chronic disease or another concomitant disease during their hospitalization (Supplementary Table 1). Newly diagnosed metabolic diseases include pre-diabetes in 21 patients (8.8%), type 2 diabetes mellitus in 14 patients (5.8%), hypertension in eleven patients (4.6%) and hyperlipidemia in eight patients (2.9%). The majority of the patients with chronic diseases were previously unaware of their diagnoses: 73.7% for diabetes mellitus, 61.1% for hypertension and 72.7% for hyperlipidemia (Table 6).

**Table 6:** Prevalence of chronic diseases.

	**All ages** **(*n* = 240)**	**20 to 29 years** **(*n* = 20)**	**30 to 39 years** **(*n* = 39)**	**40 to 49 years** **(*n* = 120)**	**≥ 50 years** **(*n* = 61)**	***P*-value**
**Pre-diabetes**						
Total, *n* (%)	21 (8.8)	1 (5.0)	2 (5.1)	9 (7.5)	9 (14.8)	0.0601
Previously known, *n* (%)	0 (0.0)	0 (0.0)	0 (0.0)	0 (0.0)	0 (0.0)	Not applicable
Newly diagnosed, *n* (%)	21 (8.8)	1 (5.0)	2 (5.1)	9 (7.5)	9 (14.8)	0.0601
**Diabetes mellitus**						
Total, *n* (%)	19 (7.9)	0 (0.0)	1 (2.6)	12 (10.0)	6 (9.8)	0.0057
Previously known, *n* (%)	5 (2.1)	0 (0.0)	0 (0.0)	4 (3.3)	1 (1.6)	0.1710
Newly diagnosed, *n* (%)	14 (5.8)	0 (0.0)	1 (2.6)	8 (6.7)	5 (8.2)	0.0329
**Hypertension**						
Total, *n* (%)	18 (7.5)	0 (0.0)	3 (7.7)	7 (5.8)	8 (13.1)	0.0071
Previously known, *n* (%)	7 (2.9)	0 (0.0)	0 (0.0)	4 (3.3)	3 (4.9)	0.0639
Newly diagnosed, *n* (%)	11 (4.6)	0 (0.0)	3 (7.7)	3 (2.5)	5 (8.2)	0.0148
**Hyperlipidemia**						
Total, *n* (%)	11 (4.6)	0 (0.0)	2 (5.1)	4 (3.3)	5 (8.2)	0.0495
Previously known, *n* (%)	3 (1.3)	0 (0.0)	0 (0.0)	2 (1.7)	1 (1.6)	0.4301
Newly diagnosed, *n* (%)	8 (3.3)	0 (0.0)	2 (5.1)	2 (1.7)	4 (6.6)	0.0411

*Data are number of patients (percentage of total number of patients in each age group). P-values are calculated using chi–squared tests*.

## Discussion

To our knowledge, this is the first analysis that investigated the clinical complications of COVID-19 and the associated burden of undiagnosed chronic disease among international migrant workers. Notably the prevalence of previously undiagnosed DM among international migrant workers is 5.8%, which is two times higher than the prevalence of undiagnosed DM among Singapore residents (2.9%) (24, 25). Even though the prevalence of hyperlipidemia (7.5 vs. 33.6%) and hypertension (5.8 vs. 21.5%) are lower in our cohort of migrant workers compared to Singapore residents, the unawareness rates are higher among migrant workers: 72.7 vs. 40.9% for hyperlipidemia, and 61.1 vs. 43.9% for hypertension (24, 25). Interestingly, our analysis shows that pre-diabetes, which is more prevalent than DM in Singapore (14.4 vs. 8.6%) (25), increases the risk of pneumonia and electrolyte abnormalities with COVID-19, indicative of more severe lung and kidney involvement. Pre-diabetes might have conferred a greater risk for pneumonia and electrolyte abnormalities than DM because, unlike patients with DM, patients with pre-diabetes were not started on pre-meal capillary blood glucose monitoring, subcutaneous sliding-scale insulin or oral hypoglycemic agents to control their blood glucose levels during their hospitalizations. Furthermore, patients with DM or pre-diabetes contract COVID-19 with lower viral RNA levels, which suggests a lower threshold for infection for both DM and pre-diabetes.

Therefore, clinicians should be encouraged to screen migrant workers for chronic diseases, in particular for DM, which increases the risk of developing severe infections, and has devastating systemic complications if not adequately treated at an early stage. Moreover, public health policies targeting migrant workers should encompass metabolic diseases, which we found to be largely undiagnosed in the migrant community compared to the resident population. For instance, most migrant workers in Singapore already incur considerable debt due to expensive fees charged by employment agencies in their home countries and are not entitled to health care subsidies afforded to Singapore residents (4). While employers of migrant workers in Singapore are required to maintain medical insurance coverage of at least $15,000 for hospitalization and day surgery costs (28), healthcare accessibility, and affordability may be improved through the extension of the mandatory insurance scheme to include preventative, primary, and outpatient care. Accessible, affordable, and appropriate heath care and insurance coverage would ensure that both chronic and acute diseases are detected and treated adequately.

Even though serious complications of COVID-19 were less prevalent in our cohort, possibly due in part to the younger demographic profile, we found significant incidental findings such as pulmonary tuberculosis, lung nodules, and pleural thickening on chest X-rays. As occupational respiratory illnesses and pulmonary tuberculosis were more prevalent among migrant workers, contributed by crowded living conditions, high stress levels, long working hours, and occupational exposure to hazardous substances (1, 4), our review suggests that screening chest X-rays might be relevant for this vulnerable population. Moreover, the COVID-19 outbreak among migrant workers also highlights the importance of improving the living and working conditions of migrant workers, to reduce the incidence of infective and occupational lung disease.

Antibiotic use was also appropriate in our study. In total, 19 of 240 (7.9%) hospitalized patients in our cohort received antibiotics for superimposed bacterial infections, which is similar to the prevalence of bacterial coinfection among hospitalized COVID-19 patients worldwide (29). Notably, a literature review of studies of hospitalized COVID-19 patients determined that while 72% of hospitalized patients received antibiotics, only 8% had evidence of superimposed bacterial or fungal coinfection (29). Responsible antimicrobial stewardship would prevent the emergence of untreatable drug-resistant pathogens that could lead to another public health emergency (30), and prompt control of blood glucose levels may prevent overuse of antibiotics by attenuating the effects of hyperglycemia on the severity of COVID-19.

This review has a number of limitations. First, our cohort of 240 migrant workers is a small subset of the population of international migrant workers in Singapore who developed COVID-19, and its demographics are limited to male migrant workers aged from 21 to 65 years with mild to moderate COVID-19 on admission. To our knowledge, this is the first analysis of COVID-19 among hospitalized international migrant workers. Hence, we are unable to compare our cohort to other cohorts of international migrant workers in Singapore or in other countries. Second, some demographic data, such as body mass index (BMI) and length of DM in patients diagnosed before admission, are not available from our data set. Third, another limitation is the lack of a similar-sized comparison group consisting of patients in the resident population. Therefore, we had to use previously published reports to compare the prevalence of chronic diseases. Fourth, since this is a retrospective review based on a brief stay in an acute hospital, and the vast majority of the patients do not have past medical records in the Singapore health system, we are unable to ascertain if some of the laboratory abnormalities are due to COVID-19 itself or an underlying chronic condition (such as thalassemia trait causing microcytosis or chronic liver disease causing transaminitis). Moving forward, a prospective cohort study that follows up on these patients' laboratory abnormalities would enable us to better understand these abnormalities. More ambitiously, a national or international database comprising both resident and migrant COVID-19 cases would enable us to determine which factors and treatments may affect health outcomes in migrant workers with COVID-19 infections.

In conclusion, this retrospective review demonstrates that there is a significant burden of undiagnosed chronic diseases, in particular metabolic diseases such as pre-diabetes and diabetes mellitus, in migrant populations. Since many of these chronic diseases benefit from early treatment, screening of asymptomatic individuals should be extended to migrant populations. Moreover, our analysis suggests that migrant workers with DM and pre-diabetes are at a higher risk of developing complications of COVID-19 despite having lower viral RNA levels, alerting clinicians to consider screening for pneumonia and electrolyte abnormalities in this vulnerable group. Importantly, this report reinforces the public health axiom that “a community is only as strong as its weakest link,” and public health policies should advocate for improved living and working conditions for neglected groups such as migrant workers, to protect the health and well-being of all.

## Data Availability Statement

The data analyzed in this study is subject to the following licenses/restrictions: Patient confidentiality (Electronic Medical Records). Requests to access these datasets should be directed to Barbara Helen Rosario, rosario.barbara.helen@singhealth.com.sg.

## Ethics Statement

The studies involving human participants were reviewed and approved by SingHealth Centralized Institutional Review Board. Written informed consent for participation was not required for this study in accordance with the national legislation and the institutional requirements.

## Author Contributions

BR, LT, SA, JT, and TO conceived and designed the study. BR, LT, SA, JT, TO, and PG collected the data. BR, LT, and SA did the data and statistical analyses. BR, LT, SA, JC, ST, AS, SH, RF, JK, TP, and WL did the data interpretation. LT and BR wrote the manuscript. All authors contributed to the article and approved the submitted version.

## Conflict of Interest

The authors declare that the research was conducted in the absence of any commercial or financial relationships that could be construed as a potential conflict of interest.
