# Supplementary material for: COVID-19 and Undiagnosed Pre-diabetes or Diabetes Mellitus Among International Migrant Workers in Singapore
Source: Front Public Health. 2020 Nov 11;8:584249. doi: 10.3389/fpubh.2020.584249 (PMC7686043; doi:10.3389/fpubh.2020.584249)
Supplement: Supplementary file 1 [file Table_1.docx]

**Supplementary Table 1: Incidence of new diagnoses made during hospitalization.**
Data are number of new diagnoses (percentage of total number of new diagnoses).

|  | **Frequency of new diagnoses (n=181)** |
| --- | --- |
| *Endocrinological* | |
| Total, n (%) | 52 (28.7) |
| Pre-diabetes, n (%) | 21 (11.6) |
| Diabetes mellitus, n (%) | 19 (10.5) |
| Hyperlipidemia, n (%) | 11 (6.1) |
| Hashimoto’s thyroiditis, n (%) | 1 (0.6) |
| *Hematological* | |
| Total, n (%) | 44 (24.3) |
| Thrombocytopenia, n (%) | 8 (4.4) |
| Leukopenia, n (%) | 8 (4.4) |
| Anemia, n (%) | 8 (4.4) |
| Polycythemia, n (%) | 4 (2.2) |
| Microcytosis, n (%) | 15 (8.3) |
| Macrocytosis, n (%) | 1 (0.6) |
| *Respiratory* | |
| Total, n (%) | 34 (18.8) |
| Pneumonia, n (%) | 20 (11.0) |
| Atelectasis, n (%) | 6 (2.8) |
| Lung nodules, n (%) | 5 (2.8) |
| Pleural thickening, n (%) | 3 (1.7) |
| *Renal* | |
| Total, n (%) | 26 (14.4) |
| Hypokalemia, n (%) | 20 (11.0) |
| Hyponatremia, n (%) | 5 (2.8) |
| Chronic kidney disease, n (%) | 1 (0.6) |
| *Cardiovascular* | |
| Total, n (%) | 15 (8.3) |
| Hypertension, n (%) | 11 (6.1) |
| Left ventricular hypertrophy, n (%) | 2 (1.1) |
| Atrial fibrillation, n (%) | 1 (0.6) |
| Atypical chest pain, n (%) | 1 (0.6) |
| *Gastroenterological* | |
| Gastro-esophageal reflux disease, n (%) | 3 (1.7) |
| *Dermatological* | |
| Total, n (%) | 2 (1.1) |
| Tinea corporis, n (%) | 1 (0.6) |
| Intertrigo, n (%) | 1 (0.6) |
| *Neurological* | |
| Total, n (%) | 2 (1.1) |
| Peripheral neuropathy, n (%) | 1 (0.6) |
| Vasovagal syncope, n (%) | 1 (0.6) |
| *Musculoskeletal* | |
| Degenerative spine, n (%) | 2 (1.1) |
| *Urological* | |
| Testicular epidydimal cyst, n (%) | 1 (0.6) |
